# Organizational wellbeing: A model of a new Apulian COVID-19 designated hospital

**DOI:** 10.3389/fpubh.2022.963315

**Published:** 2022-10-28

**Authors:** Luigi Vimercati, Enza Sabrina Silvana Cannone, Stefania Sponselli, Antonio Caputi, Giovanni Migliore, Antonio Daleno, Anna Maria Minicucci, Gabriella Milone, Lorenzo Spagnolo, Antonella Pipoli, Luigi De Maria

**Affiliations:** ^1^Section of Occupational Medicine, Interdisciplinary Department of Medicine, University of Bari, Bari, Italy; ^2^Occupational Medicine Unit, University Hospital of Bari, Bari, Italy; ^3^Hospital Direction, University Hospital of Bari, Bari, Italy; ^4^Section of Legal Medicine, Interdisciplinary Department of Medicine, University of Bari, Bari, Italy

**Keywords:** organizational wellbeing, COVID-19 hospital, health care workers, job performance, job satisfaction

## Abstract

**Background:**

Work environment characteristics have an important impact on organizational wellbeing in health care facilities. In the Apulia Region, a new COVID-19 hospital was planned, designated and built in a few weeks for the treatment of patients infected with SARS-CoV-2. To our knowledge, this hospital, together with “Fiera Hospital” in Milan, are two of the few buildings worldwide that have been converted into new health care facilities with intensive care center units to treat COVID-19 patients, and this is the first study assessing organizational wellbeing in a newly designated COVID-19 hospital.

**Aims:**

To detect and assess the strong points, criticality, and perceptions of wellbeing/discomfort of health care workers engaged in the management of the current health emergency.

**Method:**

The study was conducted on 188 health care workers, with the “Multidimensional Organizational Health Questionnaire.”

**Results:**

We found an overall positive level of organizational wellbeing. The more positive dimensions were “Collaboration between colleagues,” “Organizational efficiency” and “Room Comfort.” Conflict situations in the workplace were poorly perceived. A very low rate of absenteeism from work was also observed.

**Conclusions:**

Our results show the effectiveness of the organizational model adopted in the management of the COVID-19 hospital, especially in view of the work and emotional overload of the personnel called to face the epidemiological emergency on the frontline, which did not adversely affect the psychophysical conditions of the workers. The success of this model is related to the coexistence of all levels of care required during any type of health emergency in a single structure, paying particular attention to the architectural, functional, and procedural aspects of health care and to the so-called “humanization” of care.

## Introduction

In Italy, as in most European countries, legislation underlined the importance of ensuring organizational wellbeing, health and quality of life in the workplace.

Organizational wellbeing can be defined as “*the ability of an organization not only to be effective and productive but also to grow and develop, ensuring an adequate degree of physical and psychological wellbeing of its workers”* (1).

Several studies have shown that work environment characteristics have an important impact on organizational wellbeing in health care organizations (2–5), producing significant physical and emotional consequences for health care workers (2, 6), and affecting the quality of job performance and patient care (2, 7–9). In fact, whenever a health organization creates a working environment that encourages organizational wellbeing, workers engage in positive behaviors that contribute to improving the quality of care (2, 10).

Essentially, an organization can be described as “healthy” if its workers are fully satisfied and consider the organization to be effective and productive (1, 2, 11).

Several authors have shown that work-related stress, a lack of job satisfaction and poor organizational wellbeing may lead to issues such as absenteeism (12), a reduction in productivity, low motivation, limited expectations, a lack of commitment, and increased complaints from patients/users (11). Furthermore, several studies found that these conditions negatively affect worker health, increasing the risk of psychosomatic disorders, emotional exhaustion and burnout. In contrast, other scientific publications have indicated that in workplaces in which employees are satisfied, there are several benefits to employees' psychophysical health and they have increased positive feelings (happiness, general satisfaction, motivation and productivity) (2).

Some studies affirm that the kind of job, interprofessional relationships, the level of responsibility, an adequate level of decision-making autonomy and career development affect the level of occupational wellbeing and job satisfaction. Conversely, variables such as an authoritarian management style, inadequate planning and organization of care activities, organizational constraints, heavy workloads, interpersonal conflict and hierarchical interprofessional relationships may result in job dissatisfaction and discomfort (2, 11).

Following the advent of the health emergency resulting from the spread of the COVID-19 pandemic, health care companies had to implement rapid changes, affecting both the workload of health professionals and company organizations, resulting in a complete disruption of the work routine.

All these factors, together with the restrictions and social isolation imposed by the lockdown period and pandemic, resulted in physical and psychological damage to health workers, compromising the so-called “organizational wellness.”

Indeed, according to different scientific studies, during the pandemic, health workers have suffered disorders such as anxiety, depression, and circadian rhythm disruption (13, 14) more often than the general population, and overwork was the main factor responsible for the onset of stress and psychological discomfort (15).

Thus, health and quality of life in the workplace have become of great interest in health management, requiring an analysis of workers' needs (16) and health prevention strategies that focuses particularly on necessities that emerged during the COVID-19 pandemic. It is also important to evaluate the physical and mental states of health workers, as their health conditions may affect the effectiveness of patient treatment and protection (2).

In Bari, southern Italy, a new COVID-19 hospital was built for treatment of patients infected with SARS-CoV-2. This hospital was commissioned by the Apulia Region in collaboration with the University Hospital Policlinico of Bari and built in the pavilions of “Fiera del Levante,” a fair quartier of Bari, where an international trade fair takes place every year. To our knowledge, this hospital, together with “Fiera Hospital” in Milan, are two of the few buildings worldwide that have been converted into new health care facilities with intensive care center units to treat COVID-19 patients. This has led to the lack of studies in literature on the role and impact of new COVID-19 designated hospitals on organizational wellbeing and physical and mental states of health workers.

Therefore, the aim of this study was to detect and assess the strong points, criticality, and perceptions of wellbeing/discomfort of health professionals engaged in the management of the current health emergency in the “Fiera” COVID-19 designated hospital.

## Materials and methods

### Participants and procedure

The survey was conducted by the Complex Operative Unit of Occupational Medicine of the University Hospital of Bari, in collaboration with the Health Department of Presidium for maxi-emergencies located in the pavilions of Bari's “Fiera del Levante.”

In June 2021, an e-mail containing information about the aim of the research, the tool used, and the method and timing for the collection of questionnaires was sent to all 198 health professionals employed in the COVID-19 Hospital. We used the e-mail survey method in order to collect data on the largest population possible, in the shortest time and at the lowest cost, without taking too much time away from the health care workers involved in the fight against COVID-19. Furthermore, this survey has already been used by several public administrations.

From the 7^th^ to 14^th^ of June 2021, during the third SARS-CoV-2 epidemic wave in Apulia Region, after providing written informed consent, a total of 188 health care workers (95% adhesion rate) participants completed, on a voluntary and anonymous basis, the questionnaire received by e-mail. A total of 10 health care workers did not provide written informed consent within the deadline and were excluded from the study.

All subjects were informed that data from the research protocol would be treated in an anonymous and collective way with scientific methods and for scientific purposes in accordance with the principles of the Declaration of Helsinki. Ethical approval was not necessary because all medical and instrumental examinations were performed according to Italian law concerning the protection of workers exposed to occupational risks (D.Lgs. 81/2008).

### Measures

The detection tool used was the “Multidimensional Organizational Health Questionnaire” (MOHQ) (17). This validated questionnaire, designed by the Department of Psychology of the University “La Sapienza” of Rome, was developed to define the “health state” of an organization by analyzing the relationship between individual and workplace contexts and identifying areas that need changes to improve working conditions.

The questionnaire explores the 12 dimensions of organizational wellbeing and each of the investigated dimensions corresponds to precise items in the questionnaire (Table 1).

**Table 1:** Correspondence between “organizational wellbeing dimensions” and questionnaire items.

**Correspondence between “organizational wellbeing dimensions” and questionnaire items**		
**N**°	**Factor**	**Questions in the questionnaire**
1	Management support	24-27-29-32-33-37-39-44-50
2	Collaboration between colleagues	19-22-40-49-52-53
3	Organizational fairness	34-38-48-54
4	Organizational efficiency	17-18-20-23-26-28-30-46-47-56
5	Conflict management	21-31-41-51
6	Stress perception	25-45-55
7	Job demands	59
8	Room comfort	16
9	Job security	57-58
10	Openness to innovation	66
11	Satisfaction	61-62
12	Psychophysical disorders	64

The updated version of the questionnaire consists of 67 questions divided into eight parts. The questionnaire items included personal, environmental and work history data (questions 1 to 15), characteristics of the working environment (questions 16 to 56), workplace safety (questions 57 and 58), characteristics of the job and tolerability of assigned tasks (questions 59 and 60), feelings experienced in workplace (questions 61 to 63), psychophysical wellbeing of the worker (questions 64 and 65), openness to innovation (question 66), and suggestions to improve the work organization (question 67).

In all sections of the questionnaire, excluding the personal data and suggestions, the information was collected through a 4-point Likert measuring scale, ranging from a minimum of NEVER (score equal to 1) to a maximum of OFTEN (score equal to 4).

The Likert scale allowed us to calculate the average score for each of the 12 dimensions. Generally, a high score coincides with a positive evaluation of the dimension. The correspondence between a high score and positivity does not always occur for all dimensions. In fact, for some dimensions (conflict management, stress perception, job demands, negative indicators of satisfaction, psychophysical disorders), it was necessary to reverse the method of scoring to standardize the reading of the results. Therefore, these indicators have a negative connotation if they are perceived as very present in the organizational context.

The overall results of the whole sample are summarized into the “General Profile,” which is representative of the different values recorded in each of the organizational health dimensions. The procedure used to define the “General Profile” required the calculation of the average value for each dimension; then, the mean value of the graph for the “General profile” was obtained by summing the average score of each dimension and, subsequently, dividing it by the total number of dimensions.

The representation in a single graph (“General Profile”) allowed an immediate comparison of the dimensions. Thus, all dimensions that exceed the general average value (shown in the graph with a dotted line) represent the fields perceived by the study population as most positive in relation to organizational wellbeing. In contrast, all dimensions with a score below the general average represent the fields that the study population perceived as critical.

In this study, data analysis was performed using IBM SPSS Statistics Version 26. We used descriptive statistics (percent, mean and standard deviation) and the chi-square test for data analysis.

*P* < 0.05 were considered statistically significant.

## Results

### Sample characteristics

The overall sample consisted of 188 health care workers. The mean age was 36.8 years. Table 2 shows the general characteristics of the study population.

**Table 2:** General characteristics of the study population (n = 188).

**Factors**	**Absolute value**	**%**
**Sex**	188	100%
Female Male Not indicated	113 74 1	60.1% 39.4% 0.5%
**Age**	188	100%
<24 years 25-34 years 35-44 years 45-54 years >55 years Not indicated	30 68 33 34 22 1	16.0% 36.2% 17.5% 18.1% 11.7% 0.5%
**Qualification**	188	100%
Elementary school Junior high school High school University degree Not indicated	2 20 32 133 1	1.1% 10.6% 17.0% 70.8% 0.5%
**Civil status**	188	100%
Unmarried Married/cohabiting Separated/divorced Widowed Not indicated	96 78 10 1 3	51.1% 41.5% 5.3% 0.5% 1.6%
**Job Position**	188	100%
Health care staff Management Not indicated	136 50 2	72.3% 26.6% 1.1%
**Working time**	188	100%
Full-time Part-time Not indicated	177 9 2	94.1% 4.8% 1.1%
**Contract type**	188	100%
Fixed term Indefinite duration Not indicated	127 59 2	67,5% 31.4% 1.1%
**Length of service**	188	100%
<1 year 1-4 years 5-9 years 10-14 years 15-20 years >20 years Not indicated	27 55 28 10 25 27 16	14.4% 29.2% 14.9% 5.3% 13.3% 14.4% 8.5%

### General profile

**Figure 1:**
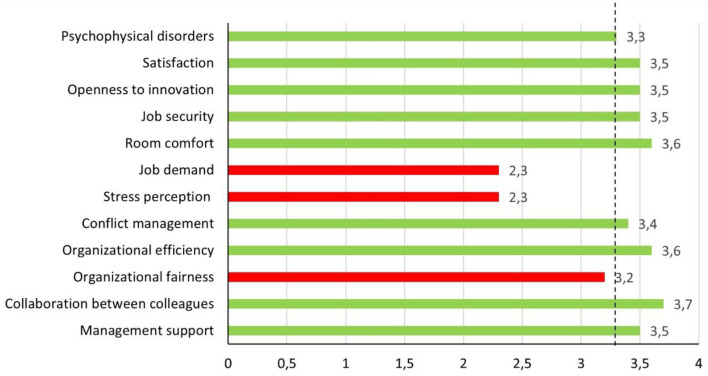
General profile. The dotted line represents the general average value of the 12 dimensions (3.3).

The general profile (Figure 1) represents the average score obtained by the whole sample in each of the 12 dimensions. It gives an indicative “picture” of organizational wellbeing. The dotted line represents the general average value of the 12 dimensions (3.3).

This value indicates a positive level of organizational wellbeing, according to the interpretative parameters suggested by the authors of the MOHQ questionnaire (Table 3).

**Table 3:** MOHQ: data interpretation criteria (17).

**Values**	**Interpretative parameters**
>2.9	Positive
2.6–2.9	Sufficient
<2.6	Negative

A histogram analysis (Figure 1) showed that the dimensions with values lower than the general average were “*Job demands*” (2.3), “*Stress perception*” (2.3), and “*Organizational fairness*” (3.2). “*Psychophysical disorders*” was in line with the average (3.3). The remaining dimensions had values that exceeded the general average; in particular, “*Collaboration between colleagues*” (3.7), “*Organizational efficiency*” (3.6) and “*Room Comfort*” (3.6) showed the highest values.

Furthermore, comparing the same dimensions with the cut-off of 2.6 suggested by the authors of the MQHQ questionnaire, only “*Job demands*” (2.3) and “*Stress perception*” (2.3) showed a negative evaluation.

### Disaggregated profiles

#### Critical dimensions

The analysis of the “*Job demands*” dimension (Table 4) showed an average value of 2.3. In particular, the factor with the lowest average value was “*Direct responsibility for work*” (1.6±0.68), followed by “*Frequent contact with people*” (1.9±0.78). In contrast, the “*Isolation*” factor showed the highest average value (3.3±0.85), comparable to the mean value of the general profile.

**Table 4:** Job demands^*^.

	**Average value ±standard deviation (SD)**
Physical strain	2.2 ± 0.86
Mental strain	2 ± 0.87
Overwork	2.5 ± 0.83
Monotony or repetitiveness	3 ± 0.83
Emotional overload	2.4 ± 1.01
Isolation	3.3 ± 0.85
Frequent contact with people	1.9 ± 0.78
Direct responsibility for work	1.6 ± 0.68
Rigid rules and procedures	2.3 ± 0.87

* For this indicator, it was necessary to reverse the method of scoring to standardize the reading of the results.

The evaluation of the connection between demographic characteristics and the perception of job demands (Table 5) showed that single civil status had a negative significant relationship with the perception of job demands (*p* < 0.038).

**Table 5:** Demographic characteristics and job demand dimension.

	**Job demand dimension**				
	<**3.3**		≥**3.3**		**Chi-square test**
	***n.***	**%**	***n.***	**%**	***p***
**Sex**					
Male (n. 74)	27	36%	47	64%	0.499
Female (n. 111)	46	62%	65	88%	
**Age (years)**					
<24 (n. 30)	15	50	15	50	0.091
25–34 (n. 67)	29	43	38	57	
35–44 (n. 33)	16	48	17	51	
45–54 (n. 33)	7	21	26	79	
>55 (n. 22)	7	32	15	68	
**Marital status**					
Single (n. 96)	47	49	49	51	0.038
Married (n. 77)	23	30	54	70	
Divorced (n. 10)	3	30	7	70	
Widowed (n. 1)	1	100	0	0	
**Contract type**					
Temporary (n. 126)	53	42	73	58	0.402
Permanent (n. 59)	21	36	38	64	
**Working time regime**					
Part-time (n. 9)	2	22	7	77	0.278
Full-time (n. 176)	71	40	105	60	
**Position Director**					
Yes (n. 50) 0	24	48	26	52	0.148
No (n. 135)	49	36	86	64	
**Working Seniority (years)**					
< 1 (n. 27)	7	26	20	74	0.109
1–4 (n. 55)	25	45	30	54	
5–9 (n. 28)	11	39	17	61	
10–14 (n. 10)	7	70	3	30	
15–20 (n. 25)	9	36	16	64	
>20 (n. 27)	7	26	20	74	

The “*Stress perception*” dimension (Table 6) showed an average value of 2.3. The analysis of the data underlines that the most critical aspect is the perception that “*The job totally consumes me*” (1.8 ± 0.82). Moreover, the other factors (“*The work tasks to be performed cause excessive fatigue*” and “*The work tasks to be performed cause excessive stress*”) also showed an average value that was lower than the general value (2.4 ± 0.8 and 2.6 ± 0.94, respectively).

**Table 6:** Stress perception^*^.

	**Average value ±standard deviation (SD)**
Excessive fatigue	2.4 ± 0.80
Excessive stress	2.6 ± 0.94
The job totally consumes me	1.8 ± 0.82

* For this indicator, it was necessary to reverse the method of scoring to standardize the reading of the results.

The evaluation of the relationship between demographic characteristics and stress perception (Table 7) showed that subjects with a length of service <1 year had the lowest perception of work-related stress (*p* < 0.011).

**Table 7:** Demographic characteristics and the stress perception dimension.

	**Stress perception dimension**				
	<**3.3**		≥**3.3**		**Chi-square test**
	***n.***	**%**	***n.***	**%**	***p***
**Sex**					
Male (n. 74)	68	92	6	8	0.986
Female (n. 112)	103	92	9	8	
**Age (years)**					
<24 (n. 30)	26	87	4	13	0.812
25–34 (n. 67)	62	92	5	8	
35–44 (n. 33)	31	94	2	6	
45–54 (n. 34)	32	94	2	6	
>55 (n. 22)	20	91	2	9	
**Marital Status**					
Single (n. 96)	88	92	8	8	0.805
Married (n. 78)	72	92	6	8	
Divorced (n. 10)	10	100	0	0	
Widowed (n. 1)	1	100	0	0	
**Contract type**					
Temporary (n. 127)	114	90	13	10	0.111
Permanent (n. 59)	57	97	2	3	
**Working time regime**					
Part-time (n. 9)	7	78	2	13	0.11
Full-time (n. 177)0	164	93	13	7	
**Position Director**					
Yes (n. 50) 0	45	90	5	10	0.557
No (n. 136)	126	93	10	7	
**Working Seniority (years)**					
< 1 (n. 27)	20	74	7	26	0.011
1–4 (n. 55)	52	94	3	5	
5–9 (n. 28)	27	96	1	4	
10–14 (n. 10)	8	80	2	20	
15–20 (n. 25)	24	96	1	4	
>20 (n. 27)	26	96	1	4	

The assessment of “*Organizational fairness*” (Table 8) showed an average value of 3.2, compared to the average value of the General Profile. However, the “*Commitment to work and personal initiatives are appreciated*” item showed a mean value of 3.4 ± 0.75, which was higher than the general average value of the 12 dimensions.

**Table 8:** Organizational fairness.

	**Average value ±standard deviation (SD)**
Career opportunities for everyone	3.1 ± 0.94
Appreciation of commitment/initiatives	3.4 ± 0.75
Opportunities for improvement	3.1 ± 0.95

In the calculation of the mean value, the item “*Economic incentives are distributed on the basis of performance efficiency*” was excluded, as only 1% of the participants responded.

For the “stress perception” dimension, the relationship between demographic characteristics and organizational fairness (Table 9) showed that subjects with a length of service <1 year had a better perception of organizational fairness (*p* < 0.011).

**Table 9:** Demographic characteristics and the organizational fairness dimension.

	**Organizational fairness dimension**				
	<**3.3**		≥**3.3**		**Chi-square test**
	***n.***	**%**	***n.***	**%**	***p***
**Sex**					
Male (n. 74)	68	92	6	8	0.986
Female (n. 112)	103	92	9	8	
**Age (years)**					
<24 (n. 30)	26	87	4	13	0.812
25–34 (n. 67)	62	92	5	8	
35–44 (n. 33)	31	94	2	6	
45–54 (n. 34)	32	94	2	6	
>55 (n. 22)	20	91	2	9	
**Marital Status**					
Single (n. 96)	88	92	8	8	0.805
Married (n. 78)	72	92	6	8	
Divorced (n. 10)	10	100	0	0	
Widowed (n. 1)	1	100	0	0	
**Contract type**					
Temporary (n. 127)	114	90	13	10	0.111
Permanent (n. 59)	57	97	2	3	
**Working time regime**					
Part-time (n. 9)	7	78	2	22	0.11
Full-time (n. 177)0	164	93	13	7	
**Position Director**					
Yes (n. 50) 0	45	90	5	10	0.557
No (n. 136)	126	93	10	7	
**Working Seniority (years)**					
< 1 (n. 27)	20	74	7	26	0.011
1–4 (n. 55)	52	94	3	6	
5–9 (n. 28)	27	96	1	4	
10–14 (n. 10)	8	80	2	20	
15–20 (n. 25)	24	96	1	4	
>20 (n. 27)	26	96	1	4	

#### Favorable dimensions

The “*Collaboration between colleagues*” dimension was the dimension with the highest average value (3.7).

Table 10 shows the average value and standard deviation for each item of this dimension. We found very high values for the items concerning the availability of workers to meet the needs of the organization and their colleagues, with values of 3.85 ± 0.36 and 3.76 ± 0.50, respectively.

**Table 10:** Collaboration between colleagues.

	**Average value ±standard deviation (SD)**
Availability to meet the needs of the organization	3.85 ± 0.36
Collaboration between colleagues	3.83 ± 0.43
Willingness to share information	3.63 ± 0.60
Efforts to achieve results	3.66 ± 0.55
Availability to meet the needs of colleagues	3.76 ± 0.50
Communications between the working group	3.43 ± 0.70
Appropriate solutions to problems	3.66 ± 0.57

*The “organizational efficiency*” dimension (Table 11) showed a mean value of 3.6. The analysis of the items highlighted positive results, especially about the presence of tools and resources to better perform the job (3.77 ± 0.5) and job satisfaction (3.73 ± 0.52).

**Table 11:** Organizational efficiency.

	**Average value ±standard deviation (SD)**
Clear and defined objectives	3.65 ± 0.62
Presence of tools/resources to better cope with the job	3.77 ± 0.50
Easily obtainable information	3.65 ± 0.58
Problem solving	3.64 ± 0.58
Satisfaction after the day's work	3.73 ± 0.52
Development of professional/individual qualities	3.53 ± 0.66
Opportunity to ask for information	3.62 ± 0.64
Satisfaction with health company initiatives	3.59 ± 0.64
Clear and well-defined work tasks	3.63 ± 0.62
Utility of services provided	3.47 ± 0.80

Table 12 represents the “*room comfort*” dimension. The assessment of the 8 factors showed average values that were constantly above the general mean value. We found that 81.4% of the workers appreciated the cleanliness and hygiene of the working rooms (average value 3.8 ± 0.49), while sanitary facilities, such as bathrooms and changing rooms, were assessed as excellent by 77.1% of the subjects (average value 3.73 ± 0.55).

**Table 12:** Room comfort.

	**Average value ±standard deviation (SD)**
Cleanliness	3.80 ± 0.49
Light	3.63 ± 0.65
Temperature	3.42 ± 0.73
Silence	3.23 ± 0.92
Building condition	3.65 ± 0.64
Pleasant rooms and furnishings	3.60 ± 0.64
Available space per worker	3.53 ± 0.74
Sanitary facilities	3.73± 0.55

Regarding the other dimensions evaluated, “*Satisfaction*” (Table 13), “*Openness to innovation*,” “*Job security*” and “*Management support*” all showed an average value of 3.5. The “*Conflict management*” (dimension had a mean value of 3.4, and “*Psychophysical disorders*” was in line with the general average value (3.3).

**Table 13:** Satisfaction (positive and negative indicators).

**Positive indicators**	**Average value ±standard deviation (SD)**	Negative indicators^*^	**Average value ±standard deviation (SD)**
Satisfaction with the organization	3.55 ± 0.67	Intolerance	3.3 ± 0.89
Desire to engage	3.67 ± 0.58	Disinterest	3.4 ± 0.80
Sense of belonging to a team	3.78 ± 0.56	Desire to change	3.3 ± 0.90
Desire to work	3.71 ± 0.56	Gossip	3.0 ± 0.99
Personal fulfillment	3.65 ± 0.61	Resentment	3.2 ± 0.95
Faith in change	3.41 ± 0.81	Aggressiveness/nervousness	3.3 ± 0.90
Work/life balance	3.39 ± 0.82	Feeling of doing useless things	3.5 ± 0.78
Excellent work relationships	3.70 ± 0.63	Feeling of being unimportant	3.1 ± 1.0
Sharing of work activities	3.64 ± 0.66	Feeling of being underestimated	3.1 ± 1.0
Faith in the leadership	3.52 ± 0.75	Inefficiency in performing tasks	3.4 ± 0.82
Faith in the morality of the leadership	3.54 ± 0.73	Dubious attribution of tasks	3.3 ± 0.9
Appreciate the job	3.46 ± 0.81	Absence of initiative	3.4 ± 0.86

* For this indicator, it was necessary to reverse the method of scoring to standardize the reading of the result.

Regarding positive indicators, “*Sense of belonging to a team*,” “*Desire to work*” and “*Excellent work relationship*” were the most expressed indicators in the study population. Regarding negative indicators, gossip was the most frequently cited factor by workers, with a mean value of 3.0, which was slightly below the general average (Table 13).

Workers perceived the working environment as open to innovation (especially regarding the introduction of new technologies and the optimization of working procedures, which results in coping with problems better) and safe.

Regarding managers, workers appreciated coherent management behaviors, their interest in work problems and their ability to treat workers fairly.

Conflict situations in the workplace were poorly perceived. In fact, approximately 79% of the workers declared the absence of psychological violence, and 127 subjects (65.4%) confirmed the absence of marginalization. Psychophysical disorders were found to be scarcely present, except for asthenia, which was often reported by 31.4% of the workers.

Finally, regarding the absences from work, we found that in the last 3 months, 84.6% of the health workers had not been absent for health reasons.

## Discussion and conclusion

The present study investigated the strong points, criticality, and perceptions of organizational wellbeing of 188 health professionals engaged in the management of the current health emergency in the recently developed Italian COVID-19 hospital in the city of Bari, highlighting the relationship between the characteristics of the work context and psychophysical wellbeing of health care workers.

In health care companies, organizational success is achieved through several factors related to the human, relational and structural aspects of the organization. Health professionals, through their expertise, must provide quality care to patients. This aspect, combined with the psychophysical wellbeing of workers, affects the effectiveness of the provision of health services (2).

Stress and dissatisfaction at work are widespread conditions in health care personnel (18). In fact, various studies have confirmed that high stress levels in health care staff are connected to lower job performance and higher absenteeism. Conversely, several studies have evaluated the conditions promoting wellbeing, motivation, and job satisfaction (19), such as clear organizational objectives, good relationships with leadership, and adequate pay and working conditions (20, 21).

The health emergency related to the spread of the COVID-19 pandemic has led to a progressive increase in the complexity of work in the health care sector, complicating relationships with people and the ability to respond to user requests and resulting in an increased assumption of direct responsibility for work. Several studies have confirmed that during the pandemic, due to hard work in very challenging conditions, health care staff members were overworked, resulting in excessive physical and psychological efforts (22, 23).

In our study, the General Profile analysis (mean value 3.3) showed a positive level of organizational wellbeing, according to the interpretative parameters indicated by the authors of the MOHQ questionnaire (Table 3). Analyzing the 12 dimensions of organizational wellbeing in detail, only three dimensions were found to be below the average calculated for the general profile, and only two of these dimensions were found to be negative with respect to the authors' interpretation criteria (Table 3).

The first critical area expressed by the sample is the so-called “Job demands” dimension. The health care workers described their jobs as intense due to the fatigue of managing daily relationships with other people, the resulting excessive sense of mental strain, the anxiety connected to direct responsibility for work, and the general sensation of feeling overworked. This dimension is the picture of a highly involved job in the cognitive and emotional spheres. Regarding the relationship between demographic characteristics and the job demand dimension, the analysis of the tested demographic variables (age, sex, contract type, working time regime, position director status and working seniority) did not show significant differences, except for marital status. In fact, the “single” workers suffered more from the burden of job demands (*p* < 0.038). This result is in line with a previous Iranian cross-sectional study that observed a negative correlation between marital status and job strain in critical care nurses (24). This result may be explained by the possibility of sharing work problems with a partner and receiving emotional support.

The mean scores analysis of the single items of “*Stress perception*” confirmed the intensity of the work as the main critical area: health workers described their job duties as stressful and exhausting. It follows that the work is often perceived as all-encompassing, as stated by 40% of the subjects. The statistical analysis highlighted a lower stress perception and a perception of better organizational fairness in subjects with less working seniority (*p* < 0.011). Research from the University of Nottingham showed how work-related stress tends to increase with age, peaking between the ages of 50 and 55 years. In fact, older workers have greater difficulties adapting to change, partly due to health problems and family responsibilities (25).

However, regarding the direct question being useful in quantifying the burden resulting from the characteristics of their work, 77.7% of the employees reported that overworking did not cause difficulties.

Regarding the third critical dimension (“*Organizational fairness*”), the three assessment factors (“*Career opportunities for everyone*,” “*Appreciation of commitment/initiatives*,” “*Opportunities of improvement*”) had no significant deviation from the mean value of the “General profile.”

Therefore, these factors assess subjective perceptions of slight discomfort. In addition, the evaluation of the “*Appreciation of commitment/initiatives*” factor showed that more than half (57%) of the workers considered their commitment to work and personal initiatives was fully appreciated. Furthermore, the evaluation of the “*Organizational Efficiency*” dimension showed excellent results. In particular, the “*Development of professional/individual qualities*” factor had an average score of 3.53, and 76.1% of the workers said they felt fully satisfied at the end of the working day. As further confirmation, the “*Openness to innovation*” dimension highlighted the ability of the organization to enhance the development of new skills in workers, as well as the willingness to introduce new professional figures to staff.

The dimensions that evaluated interactions with colleagues had the highest score (3.7). Eighty-five percent of the workers confirmed an optimal collaboration with colleagues (3.83±0.43), which was perceived as a source of work support and affective support. In the same way, management support was favorable (3.5), mainly due to the involvement shown by the managers toward the problems of the staff, their consistent behaviors and their fair treatment of workers. Approximately 66.5% of the participants reported the absence of conflicts with their superiors. To confirm how positive human interactions at all levels in the working environment are beneficial to the psychophysical health of workers, an analysis of positive indicators revealed the workers' perceptions of excellent working relationships, the sense of belonging to the team, the desire to go to work, personal satisfaction, and the sharing of work activities. The scientific and medical literature shows consistent findings about the importance of positive relationships between colleagues in the workplace. Tran et al., in a survey of 303 hospital nurses, demonstrated a lower level of job stress and higher commitment in a working environment characterized by good relationships between colleagues; in addition, the results of this study showed that high-quality relationships between leaders and their staff improved job performance (26). The importance of a positive working environment was confirmed by a recent study in which Rasool et al. suggested that a toxic work environment had a negative impact on employee engagement, spreading negative feelings among colleagues. Feelings arising from a toxic work environment (e.g., harassment, bullying or ostracism) can lead to excessive stress, burnout and anxiety among workers. The same authors showed that when workers perceived support from the organization, they increased their engagement and their sense of belonging to the organization was enhanced (27).

In our study, the job satisfaction expressed by the participants was confirmed by the good results obtained in the evaluation of negative indicators and psychophysical disorders manifested in the last 3 months. In a recent review about the impact of COVID-19 on the mental health of health care workers, De Kock et al. highlighted that high levels of stress and anxiety have been shown to decrease staff morale, increase absenteeism, and cause lower levels of work satisfaction and quality of care (28).

In our sample, asthenia, sleep disturbance and anxiety were the most frequent problems, affecting only 6.9, 11.2, and 6.9% of the sample, respectively. This state of wellbeing was confirmed by the data about absences from work: in the last 3 months, 84.6% of the health workers had not been absent for health reasons.

Regarding the characteristics of the working environment, it has long been known that the physical environment of a health care facility can affect patients and staff. In a review of the physical characteristics of the indoor environment in health care facilities, the authors suggested that the acoustic environment, ventilation system, air conditioning system, thermal environment, visual environment (e.g., lighting and views of nature), ergonomic conditions and furniture have beneficial effects for the wellbeing of patients and staff (29). In our study, the “*Room comfort*” dimension had a mean score of 3.6, followed by the “*Job security*” dimension (3.5). Most of the sample (81.4%) had a very positive view of the cleanliness of the working environment, the condition of the building (71.3%) and the lighting (70.7%). A total of 86.7% of the participants perceived a high involvement of the organization in health and safety in the workplace.

The assessment of organizational wellbeing allowed us to obtain an overall picture of the working atmosphere and to indicate important strategies to improve it and the services provided.

On the basis of the useful suggestions provided by the interviewed staff to improve organizational wellbeing, the first intervention to be put in place is the valorisation of the staff (47.3%), followed by the identification of an incentive distribution system that can reward staff for the work performed (31.9%).

Our study had certain limitations. First, the questionnaire used did not allow us to stratify the result according to the professional category of health care workers. Second, the assessment of individual psychological characteristics is lacking, although it is an important element in fully characterizing wellbeing in the workplace. It would therefore be advisable for future studies to focus on this aspect. Despite these limitations, these results could help to define and structure paths for change and training activities for workers to improve the work context. In particular, from the analysis of the identified criticalities and from the considerations of the workers, various operating strategies were derived:

- Enhancement of personnel and meritocracy, allowing operators to carry out an activity for which they have been trained, in which their performance is the best possible and the incentives are proportionate to the results achieved.
- Evaluation sheets to redistribute assignments by skills.
- “Internal working model” seminars through which a worker can face and solve stressful events.
- Improvement of procedures to facilitate the performance of demanding activities.
- Early detection and intervention of risk situations (i.e., stress, burnout).
- Training activities.
- Focus groups for sharing critical issues connected to emotional experiences that are related to work.
- Optimizing work and life balance, managing working time in a more flexible way, aiming at the result and not at the number of working hours.

Our results show the effectiveness of the organizational model adopted in the management of the COVID-19 hospital in Bari, especially in view of the work and emotional overload of the personnel called to face the epidemiological emergency on the frontline, which, however, did not adversely affect the psychophysical conditions of the workers.

The success of this model is related to the coexistence of all levels of care (medical, surgical, services) required during any type of health emergency in a single structure. Moreover, the importance of paying particular attention to the architectural, functional, and procedural aspects of health care and to the so-called “humanization” of care has been realized. All these factors show that the place where therapies are carried out can influence outcomes, helping to improve the job performance of health workers and the psychophysical conditions of patients.

## Data availability statement

The data presented in this study are available on request from the corresponding author.

## Ethics statement

Ethical approval was not provided for this study on human participants because Ethical Approval is not necessary because all the medical and instrumental examinations were performed according to the Italian laws concerning the protection of workers exposed to occupational risks (D. Lgs. 81/2008). The authors assert that all procedures contributing to this work comply with the ethical standards of the relevant national and institutional committees on human experimentation and with the Helsinki Declaration of 1975, as revised in 2008. All procedures involving human subjects/patients have been approved by the Occupational Medicine Unit, which deals with the health surveillance of all health care workers in the hospital, in accordance with the Italian legislation on the protection of workers exposed to occupational risks (Legislative Decree n.81/2008). The patients/participants provided their written informed consent to participate in this study.

## Author contributions

LV, GMig, AD, AM, and GMil conceived and designed the study. LD, EC, SS, AC, and LS were responsible for the acquisition and analysis of the data. AP performed the statistical analyses. LD, EC, SS, and AC wrote the paper. All authors approved the final version.

## Conflict of interest

The authors declare that the research was conducted in the absence of any commercial or financial relationships that could be construed as a potential conflict of interest.

## Publisher's note

All claims expressed in this article are solely those of the authors and do not necessarily represent those of their affiliated organizations, or those of the publisher, the editors and the reviewers. Any product that may be evaluated in this article, or claim that may be made by its manufacturer, is not guaranteed or endorsed by the publisher.

## References

[1] Avallone F Bonaretti M. Benessere organizzativo. Soveria Mannelli: Rubbettino Editore. (2003).

[2] Zaghini F Vellone E Maurici M Sestili C Mannocci A Ercoli E. The influence of work context and organizational wellbeing on psycophysical health of healthcare providers. Med Lav. (2020) 111:306–20. 10.23749/mdl.v111i4.9075 32869767 PMC7809958

[3] Jain R Kaur S. Impact of work environment on job satisfaction. Int J Sci Res. (2014) 4:1–8. Available online at: https://www.ijsrp.org/research-paper-0114.php?rp=P25221234512206

[4] Khamisa N Oldenburg B Peltzer K Ilic D. Work related stress, burnout, job satisfaction and general health of nurses. Int J Environ Res Public Health. (2015) 12:652–66. 10.3390/ijerph120100652 25588157 PMC4306884

[5] Vimercati L Tafuri S Chironna M Loconsole D Fucilli FIM Migliore G. The COVID-19 hotel for healthcare workers: an Italian best practice. J Hosp Infect. (2020) 105:387–8. 10.1016/j.jhin.2020.05.018 32425286 PMC7229477

[6] Shamian J Kerr MS Laschinger HK Thomson D. A hospital-level analysis of the work environment and workforce health indicators for registered nurses in Ontario's acute-care hospitals. Can J Nurs Res. (2016) 33:35–50. 11998196

[7] Kieft RA de Brawer BBJM Francke AL Delnoij DM. How nurses and their work environment affect patient experiences of the quality of care: a qualitative study. BMC Health Serv Res. (2014) 14:249. 10.1186/1472-6963-14-249 24923663 PMC4064111

[8] Vimercati L De Maria L Quarato M Caputi A Stefanizzi P Gesualdo L. COVID-19 hospital outbreaks: Protecting healthcare workers to protect frail patients. An Italian observational cohort study. Int J Infect Dis. (2021) 102:532–7. 10.1016/j.ijid.2020.10.098 33157297 PMC7610093

[9] Boffetta P Violante F Durando P De Palma G Pira E Vimercati L. Determinants of SARS-CoV-2 infection in Italian healthcare workers: a multicenter study. Sci Rep. (2021) 11:5788. 10.1038/s41598-021-85215-4 33707646 PMC7970984

[10] Montgomery A Spânu F Băban A Panagopoulou E. Job demands, burnout and engagement among nurses: a multi-level analysis of ORCAB data investigating the moderating effect of teamwork. Burn Res. (2015) 2:71–9. 10.1016/j.burn.2015.06.001 26877971 PMC4710673

[11] Sili A Vellone E De Marinis MG Fida R Venturini G Alvaro R. Validity and reliability of the nursing organizational health questionnaire. Prof Inf. (2010) 63:27–37. Available online at: https://www.profinf.net/pro3/index.php/IN/article/view/27 20470457

[12] Diestel S Wegge J Schmidt KH. The impact of social context on the relationship between individual job satisfaction and absenteeism: the roles of different foci of job satisfaction and work-unit absenteeism. Acad Manag J. (2014) 57:353–82. 10.5465/amj.2010.1087

[13] Lai J Ma S Wang Y Cai Z Hu J Wei N. Factors associated with mental health outcomes among health care workers exposed to coronavirus disease 2019. JAMA Netw Open. (2020) 3:e203976. 10.1001/jamanetworkopen.2020.3976 32202646 PMC7090843

[14] Liang Y Wu K Zhou Y Huang X Zhou Y Liu Z. Mental health in frontline medical workers during the 2019 novel coronavirus disease epidemic in China: a comparison with the general population. Int J Environ Res Public Health. (2020) 17:6550. 10.3390/ijerph17186550 32916836 PMC7558595

[15] Zerbini G Ebigbo A Reicherts P Kunz M Messman H. Psychosocial burden of healthcare professionals in times of COVID-19 - a survey conducted at the University Hospital Augsburg. Ger Med Sci. (2020) 18:Doc05. 10.3205/000281 32595421 PMC7314868

[16] Shanafelt T Ripp J Trockel M. Understanding and addressing sources of anxiety among health care professionals during the COVID-19 pandemic. JAMA. (2020) 323:2133–4. 10.1001/jama.2020.5893 32259193

[17] Avallone F Paplomatas A. Salute Organizzativa, psicologia del benessere nei contesti lavorativi. Raffaello Cortina Editore: Milano. (2005).

[18] de Sousa CC de Araújo TM Lua I Gomes MR. Occupational stress and job dissatisfaction with health work. Psicol Reflex Crit. (2019) 32:18. 10.1186/s41155-019-0132-5 32026001 PMC6966978

[19] Bakker AB Demerouti E. Job demands-resources theory: taking stock and looking forward. J Occup Health Psychol. (2017) 22:273–85. 10.1037/ocp0000056 27732008

[20] Chamberlain SA Hoben M Squires JE Estabrooks CA. Individual and organizational predictors of health care aide job satisfaction in long term care. BMC Health Serv Res. (2016) 16:577. 10.1186/s12913-016-1815-6 27737672 PMC5064796

[21] Aloisio LD Gifford WA McGilton KS Lalonde M Estabrooks CA Squires JE. Individual and organizational predictors of allied healthcare providers' job satisfaction in residential long-term care. BMC Health Serv Res. (2018) 18:491. 10.1186/s12913-018-3307-3 29940949 PMC6019323

[22] Jeleff M Traugott M Jirovsky-Platter E Jordakieva G Kutalek R. Occupational challenges of healthcare workers during the COVID-19 pandemic: a qualitative study. BMJ Open. (2022) 12:e054516. 10.1136/bmjopen-2021-054516 35256442 PMC8905413

[23] WHO. Keep Health Workers Safe to Keep Patients Safe. Geneva. (2020). Available online at: https://www.who.int/news/item/17-09-2020-keep-health-workers-safe-to-keep-patients-safe-who (accessed on 18th October 2022)

[24] Vahedian-Azimi A Hajiesmaeili M Kangasniemi M Fornés-Vives J Hunsucker RL Rahimibashar F. Effects of stress on critical care nurses: a national cross-sectional study. J Intensive Care Med. (2019) 34:311–22. 10.1177/0885066617696853 29277137

[25] Griffiths A Knight A Mahudin DNM. Ageing, Work Related Stress Health. Reviewing the evidence. A report for Age Concern Help the Aged, TAEN - The Age Employment Network. Institute of Work, Health Organisations, University of Nottingham (2009). Available online at: https://www.dors.it/documentazione/testo/201603/Work_Related_Stress_32pg.pdf (accessed October 18, 2022).

[26] Tran KT Nguyen PV Dang TTU Ton TNB. The impacts of the high-quality workplace relationships on job performance: a perspective on staff nurses in Vietnam. Behav Sci. (2018) 8:109. 10.3390/bs8120109 30477199 PMC6316783

[27] Rasool SF Wang M Tang M Saeed A Iqbal J. How toxic workplace environment effects the employee engagement: the mediating role of organizational support and employee wellbeing. Int J Environ Res Public Health. (2021) 18:2294. 10.3390/ijerph18052294 33652564 PMC7956351

[28] De Kock JH Latham HA Leslie SJ Grindle M Munoz SA Ellis L. A rapid review of the impact of COVID-19 on the mental health of healthcare workers: implications for supporting psychological wellbeing. BMC Public Health. (2021) 21:104. 10.1186/s12889-020-10070-3 33422039 PMC7794640

[29] Salonen H Lahtinen M Lappalainen S Nevala N Knibbs L Morawska L. Physical characteristics of the indoor environment that affect health and wellbeing in healthcare facilities: *A review*. Intell Build Int. (2013) 5:3–25. 10.1080/17508975.2013.764838

